# Emerging insights into Panton-Valentine leukocidin-producing methicillin-resistant *Staphylococcus aureus* in Gulf Cooperation Council countries: A scoping review

**DOI:** 10.5339/qmj.2026.63

**Published:** 2026-09-17

**Authors:** Sreethish Sasi, Ali Senosi Omrani, Theresa Paul, P Chitrambika, Muna Al Maslamani

**Affiliations:** 1 Infectious Diseases Division, Department of Internal Medicine, Communicable Diseases Center, Hamad Medical Corporation, Doha, Qatar; 2 Faculty of Medicine, Qatar University, Doha, Qatar; 3 Montefiore Medical Centre, Wakefield Campus, Bronx, New York, USA; 4 Department of Anesthesiology, Hamad Medical Corporation, Doha, Qatar

**Keywords:** MRSA, PVL, Review, GCC, Qatar

## Abstract

**Background:**

Panton-Valentine leukocidin (PVL)-producing methicillin-resistant *Staphylococcus aureus* (MRSA) is a growing public health concern in Gulf Cooperation Council (GCC) countries. PVL enhances MRSA virulence, contributing to severe skin and soft tissue infections and necrotizing pneumonia.

**Objectives:**

To review the prevalence, molecular characteristics, and antibiotic resistance patterns of PVL-positive MRSA strains in GCC countries, i.e., Bahrain, Kuwait, Oman, Qatar, Saudi Arabia, and the United Arab Emirates (UAE).

**Methods:**

We conducted a scoping review of studies published between January 2020 and December 2025 following the Preferred Reporting Items for Systematic Reviews and Meta-Analyses extension for Scoping Reviews (PRISMA-ScR) guidelines. Searches were performed in PubMed, EMBASE, Scopus, Cochrane Library, and Google Scholar, targeting original research reporting PVL presence in clinical MRSA isolates from GCC countries. Data were extracted into standardized forms and organized in comparative tables.

**Results:**

Fourteen original studies were included, primarily from Saudi Arabia, Kuwait, UAE, Bahrain, and Qatar. Across included studies, PVL prevalence among MRSA isolates varied widely, ranging from 0% to 62% depending on country, setting, and study design. Community-associated (CA) MRSA clones, such as ST80-IV, ST30-IV, and ST6-IV, predominated among PVL-positive strains, though PVL acquisition by hospital-associated (HA) lineages like ST22-IV and ST239-III was increasingly reported. PVL-positive strains exhibited resistance to fusidic acid, fluoroquinolones, and macrolides, but remained susceptible to vancomycin and linezolid.

**Conclusion:**

PVL-producing MRSA is a significant contributor to staphylococcal disease in the GCC, with prevalence in both community and hospital settings. The convergence of virulence and multidrug resistance among PVL-MRSA strains presents challenges for infection control. Strengthened regional surveillance, standardized molecular typing, and one-health-informed monitoring are needed.

## 1. INTRODUCTION 

Methicillin-resistant *Staphylococcus aureus* (MRSA) represents a persistent global health challenge.^1^ MRSA infections range from mild skin conditions to severe pneumonia, bacteremia, and device-related infections.^2^ Among MRSA virulence factors, Panton–Valentine leukocidin (PVL) is important due to its role in leukocyte destruction and tissue necrosis.^3^ The PVL genes (*lukS-PV* and *lukF-PV*) are commonly found in community-acquired (CA)-MRSA lineages and have been implicated in outbreaks of skin abscesses and necrotizing pneumonia.^4^ PVL-positive MRSA strains gained attention with the emergence of CA-MRSA infections among healthy individuals.^5^ While CA-MRSA strains, such as USA300 and EUST80, were initially more antibiotic-susceptible than traditional hospital-acquired (HA)-MRSA, the distinction between these categories has blurred.^3,6^ Genetic exchange and clonal spread have enabled PVL-positive strains to infiltrate healthcare settings.^7,8^

MRSA accounts for 15% to 55% of S. aureus infections in Gulf Co-operation Council (GCC) countries.^1^ MRSA among *Staphylococcus aureus* isolates in the United Arab Emirates (UAE) increased from 21.9% in 2010 to 33.5% in 2021.^7^ CA-MRSA generally describes strains arising outside traditional healthcare settings and has historically been linked to enhanced virulence and lower resistance burdens, whereas HA-MRSA has typically been associated with healthcare exposure and multidrug resistance.^6^ Nevertheless, these categories are no longer sharply separated, as CA clones are increasingly encountered in hospitals and HA clones continue to circulate more broadly.^7^ A large proportion of MRSA in the region belongs to PVL-positive CA lineages.^9–11^ PVL genes are generally absent in classic HA-MRSA strains with minimal community exposure (e.g., 0% PVL detection among 83 HA-MRSA isolates in Medina, Saudi Arabia).^11^ However, this pattern is changing. CA-MRSA clones, such as ST80-IV (EUST80), ST30-IV (USA1100), and ST6-IV (WA-MRSA-51), are now frequently encountered in hospitals.^1^ Meanwhile, traditional HA-MRSA clones like ST22-IV (UK-EMRSA-15) and ST239-III continue to circulate in healthcare settings.^7,12–14^ Some of them are acquiring PVL and other mobile genetic elements previously associated with CA strains. PVL-positive MRSA has been linked to more severe clinical presentations, including recurrent abscesses, necrotizing infections,^15^ and toxic shock syndrome toxin-1 (TSST-1).^6^ Increasing detection of multidrug resistance among PVL-positive MRSA compounds the problem.^6^

The GCC region merits a dedicated review because its members share several structural, demographic, and epidemiologic characteristics relevant to the circulation of MRSA.^1^ High expatriate mobility, frequent travel between neighboring countries, interconnected healthcare systems, comparable hospital and referral pathways, and similar antimicrobial access and prescribing practices together create a setting in which successful resistant and virulent clones may emerge and disseminate across national boundaries.^1^ In addition, a One-Health perspective, which emphasizes the interdependence of human, animal, food, and environmental health, is important in this region for understanding the broader reservoirs and transmission ecology of MRSA.^2^ PVL was chosen as the focus of this review because it is among the most frequently reported virulence markers in regional CA-MRSA literature and provides a useful lens to examine the evolving overlap between virulence and antimicrobial resistance in GCC countries.^15^ This review analyzes PVL-positive MRSA prevalence, molecular traits, and resistance profiles in GCC countries from 2020–2025, aiming to guide infection control and public health policies.

## 2. MATERIALS AND METHODS

### 2.1. Study design

This scoping review was reported in accordance with the Preferred Reporting Items for Systematic Reviews and Meta-Analyses extension for Scoping Reviews (PRISMA-ScR). The review focused on studies published between 1 January 2020 and 31 December 2025 and included evidence from Bahrain, Kuwait, Oman, Qatar, Saudi Arabia, and the UAE. Although the primary emphasis was on human clinical epidemiology, selected non-clinical studies involving food or meat sources were considered eligible when they contributed directly to the One-Health context of MRSA circulation in the region. These non-clinical studies were included to provide contextual evidence regarding possible overlap between human and non-human reservoirs and were not weighted equally with human clinical studies in the interpretation of findings.

### 2.2. Search strategy

A structured literature search was performed in PubMed on 20 January 2026, Embase on 22 January 2026, Scopus on 25 January 2026, Cochrane Library on 28 January 2026, and Google Scholar on 2 February 2026. The search strategy combined controlled vocabulary terms, where applicable, and free-text keywords related to MRSA, PVL, and GCC countries. The core Boolean search strategy was as follows: ("methicillin-resistant *Staphylococcus aureus*" OR MRSA OR "*Staphylococcus aureus*") AND ("Panton-Valentine leukocidin" OR PVL OR "lukS-PV" OR "lukF-PV" OR leukocidin) AND ("Gulf Cooperation Council" OR GCC OR Bahrain OR Kuwait OR Oman OR Qatar OR "Saudi Arabia" OR "United Arab Emirates" OR UAE). Database-specific adjustments were made as needed. In PubMed, medical subject headings and title or abstract terms were used where appropriate. Searches were limited to English-language publications published from January 2020 to December 2025. Reference lists of relevant articles were also manually screened to identify any additional eligible studies. For Google Scholar, the search was restricted to the first 250 records, sorted by relevance, to ensure feasibility and reproducibility. Duplicate citations across databases were removed before screening.

### 2.3. Eligibility criteria

Studies were eligible for inclusion if they met all of the following criteria:

1. They reported original data from one or more GCC countries.
2. They investigated PVL genes, PVL-positive MRSA isolates, or both.
3. They were published between January 2020 and December 2025.
4. They were original research articles with analyzable epidemiologic, microbiologic, molecular, or resistance-related data.

Both CA-MRSA and HA-MRSA studies were eligible. Studies from non-clinical sources, including food or meat, were included only when they were relevant to the review’s One-Health objective and could inform possible epidemiologic overlap with human strains.

Studies were excluded if they:

1. Did not include data from GCC countries.
2. Discussed MRSA without reporting any PVL-related findings.
3. Were review articles, editorials, correspondence, or single case reports without broader analyzable data.
4. Were not published in English.
5. Were purely animal or environmental studies without direct relevance to the human or food-related One-Health context addressed in this review.

### 2.4. Study selection 

**Figure 1.**
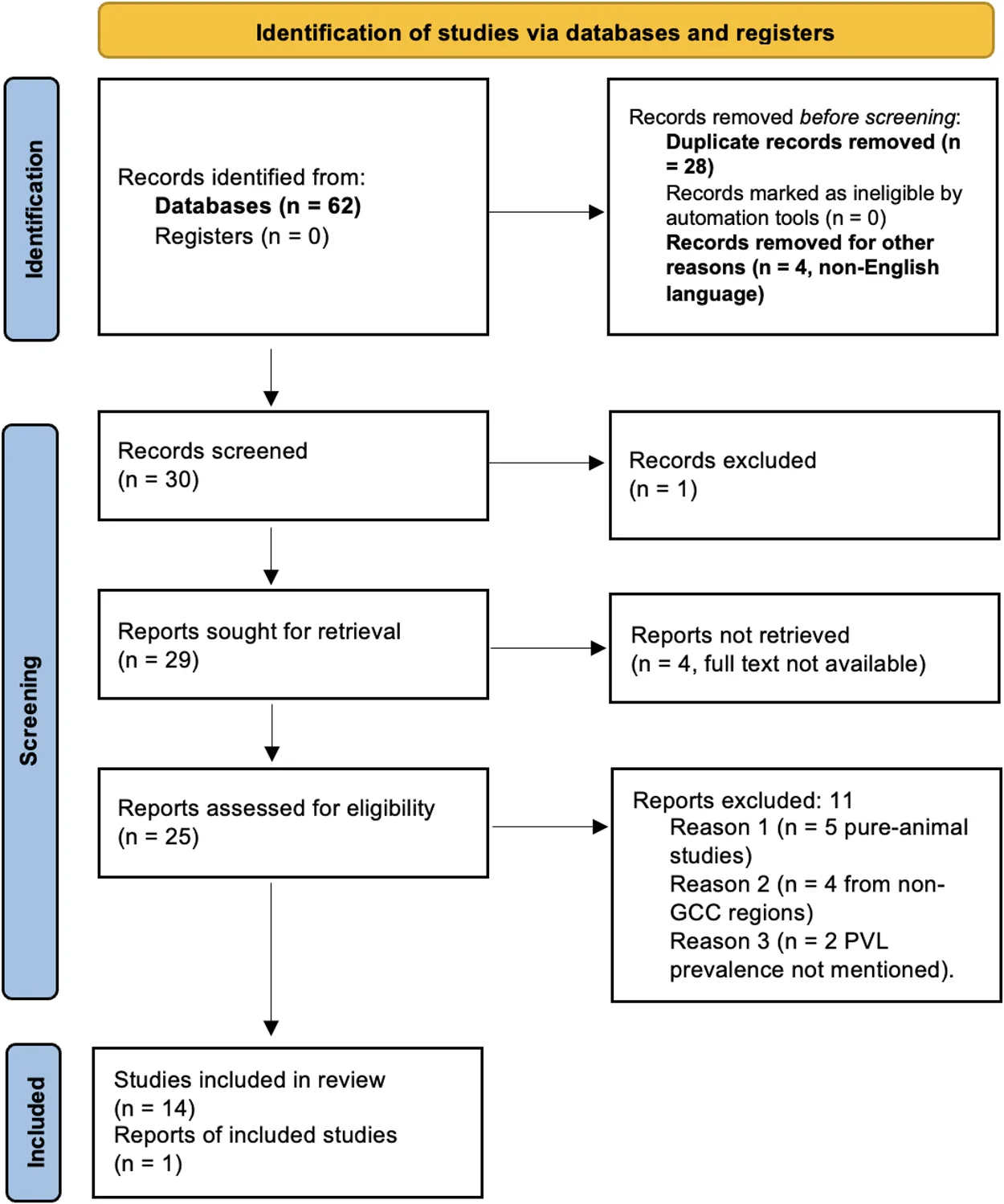
PRISMA-ScR flow diagram of study selection for the scoping review.

All retrieved records were imported into rayyan.ai (AI-Powered Systematic Review Management Platform) for study management and duplicate removal. A total of 62 records were identified through database searches and reference screening. After removal of duplicates, 30 unique records remained for title and abstract screening. Of these, 25 articles were considered potentially eligible and underwent full-text review. Following full-text assessment, 11 studies were excluded for the following reasons: lack of PVL-specific results (*n* = 2), absence of GCC-specific data (*n* = 4), and purely animal studies without direct relevance to the scope of this review (*n* = 5). A final total of 14 studies met the eligibility criteria and were included in the review. Title or abstract screening and full-text assessment were performed independently by two reviewers ([SS] [CPKM]). Disagreements were resolved through discussion and consensus; where needed, a third reviewer ([TP]) adjudicated unresolved discrepancies. The study selection process is illustrated in the PRISMA-ScR flow diagram (Figure 1).

### 2.5. Data charting and synthesis

Data were extracted using a standardized data-charting form developed for this review. For each included study, the following information was collected: author and year, country, study setting, study design, study period, sample size, sample source, prevalence of PVL-positive MRSA, dominant clones or sequence types, staphylococcal cassette chromosome mec types, spa types where available, antimicrobial resistance (AMR) patterns, other relevant virulence markers, and the main conclusions of the study.

The data were synthesized descriptively and summarized in comparative Tables (i.e., Tables 1–3). Because the included studies were heterogeneous in design, setting, patient population, sampling approach, and reported outcomes, the review did not attempt quantitative pooling or meta-analysis. Prevalence findings are therefore presented as study-level ranges rather than as a formal regional pooled estimate.

### 2.6. Critical appraisal 

A formal risk-of-bias or methodological quality assessment was not performed. This decision was made because the present study was designed as a scoping review, whose purpose was to map the breadth, characteristics, and heterogeneity of the available evidence rather than to generate pooled effect estimates or comparative causal inferences. This lack of formal quality appraisal is acknowledged as a limitation of the review.

## 3. RESULTS

A total of 14 original research articles were included in this review. Seven studies originated from Saudi Arabia, three from Kuwait, two from the UAE, and one each from Bahrain and Qatar. Notably, no eligible original studies from Oman were identified during the review period, highlighting an important evidence gap in the regional literature and limiting the completeness of GCC-wide epidemiologic interpretation (Table 1).

### 3.1. Prevalence of PVL-positive MRSA

PVL prevalence among MRSA isolates varied substantially across studies and countries, ranging from 0% in strictly hospital-acquired cohorts^11^ to over 60% in community-rich hospital settings.^8,9^ In Bahrain, 62% of MRSA isolates were PVL-positive,^8^ while a hospital in Jeddah, Saudi Arabia, reported 60% positivity.^9^ In the UAE, a comprehensive genomic survey found a 36% PVL prevalence among clinical MRSA.^7^ A multicenter Saudi study reported 46% PVL positivity in hospital infections.^14^ Conversely, in a healthcare-associated MRSA cohort from Medina, no PVL-positive isolates were identified.^11^ Surveillance studies highlighted that PVL was more common in community-acquired cases than in hospital-acquired ones. In Bahrain, 66% of CA-MRSA carried PVL compared to 33% in HA-MRSA.^8^ Similar patterns were observed in Saudi Arabia^9^ and Kuwait (Table 2).^12^

### 3.2. Dominant MRSA clones

The most commonly reported PVL-positive clones were ST80-IV, ST30-IV, and ST6-IV. ST80-IV was widespread across Kuwait^12^ and Saudi Arabia,^11,14,16^ while ST30-IV predominated in Qatar^17^ and parts of the UAE.^7^ ST6-IV was prominent in Oman^18^ and present in multiple countries. USA300 (ST8-IV) and USA400 (ST1-IV) clones were rare but detected in the UAE.^7,18^ Hospital-associated clones, such as ST22-IV (CC22) and ST239-III, remain prevalent, but an increasing number now carry PVL. Notably, a PVL-positive and TSST-1–positive ST22-IV variant has emerged in Kuwait,^12,13^ and Saudi Arabia.^11,14,16^ Other noteworthy clones include CC1153, which consistently carries PVL and was identified in the UAE and Saudi Arabia, and CC361, which has been implicated in Kuwaiti hospital outbreaks. Though typically PVL-negative, CC361 has recently been identified in PVL-positive forms in the UAE. Overall, the clonal landscape in the GCC is diverse and dynamic, with high strain heterogeneity in the UAE and Bahrain and more stable clonal dominance (e.g., ST22-IV, CC361) in Kuwait.

### 3.3. Antibiotic resistance patterns

PVL-positive MRSA strains commonly exhibited resistance to fusidic acid, fluoroquinolones, macrolides, and clindamycin. Fusidic acid resistance was linked to the presence of fusC, often embedded in composite SCC elements.^19^ In Kuwait, over three-quarters of multidrug-resistant ST22 isolates were PVL-positive, some co-carrying TSST-1.^12,13^ Ciprofloxacin resistance was frequent in Bahrain, Kuwait, and Saudi Arabia, particularly among PVL-positive clones. Despite the multidrug resistance, vancomycin and linezolid susceptibility was preserved across all studies. Resistance to trimethoprim-sulfamethoxazole and tetracyclines varied but was generally lower than for other agents (Table 3).

### 3.4. Settings and sample sources

The majority of studies (12 of 14) analyzed clinical MRSA isolates from hospitals, encompassing both community and hospital-onset cases. In some settings, over 80% of hospital MRSA cases were community-associated. Only two studies focused on non-hospital community settings: One in Qatar reported no PVL in nasal and axillary carriage among 576 participants;^17^ another compared clinical isolates with MRSA from retail meats.^20^ Three studies examined non-human or food-related MRSA, highlighting limited PVL presence in meat isolates.^20–22^ However, clonal overlap with human strains (notably ST5 and ST6) was observed, suggesting potential bidirectional transmission. These findings emphasize the importance of One-Health surveillance.

## 4. DISCUSSION

### 4.1. Regional epidemiology and shifting trends

PVL-positive MRSA is a significant public health concern in GCC countries, with studies showing high rates, especially in community-onset infections. While prevalence rates vary by setting and country, multiple studies found PVL in over half of MRSA isolates.^8,9,14,16,20^ Such findings reflect the rise of virulent community MRSA strains in hospital environments. Distinct lines between CA- and HA-MRSA are increasingly blurred in the GCC. CA clones have infiltrated hospitals, and in some centers, they now dominate. For example, CA-MRSA constituted 88% of MRSA infections in a Bahrain hospital,^8^ and similar findings were reported from Saudi hospitals.^11,14,16^ Concurrently, hospital-adapted clones have begun acquiring PVL and other community-associated features, resulting in hybrid lineages with both high virulence and multidrug resistance.

### 4.2. Clonal diversity and evolution

The clonal composition of PVL-positive MRSA in the GCC reflects both global dissemination and regional evolution. ST80, ST30, and ST6 dominate among CA-MRSA strains, while ST22 and ST239 persist in hospitals, with emerging PVL-positive variants. ST22 in particular has evolved in Kuwait and Saudi Arabia into hypervirulent, multidrug-resistant clones co-harboring PVL and TSST-1.^10,13,20,22^ Emerging lineages such as CC1153 and CC361 are of special interest. CC1153 is uniquely PVL-positive, fusidic acid–resistant and appears region-specific. CC361, previously unreported in other regions, has become a dominant hospital strain in Kuwait and recently acquired PVL in UAE isolates, indicating ongoing genetic exchange.^7,13^ The UAE and Bahrain exhibit greater clonal heterogeneity, likely due to international travel and population diversity.^7,8^ Conversely, Kuwait’s dominance of specific clones, such as CC22 and CC361, reflects a more stable but evolving hospital epidemiology.^13^

### 4.3. Antibiotic resistance concerns

PVL-positive MRSA strains in the GCC are not uniformly susceptible to non-beta-lactam antibiotics, contrary to earlier assumptions about CA-MRSA. Fusidic acid resistance, driven by fusC, is widespread, possibly driven by the anecdotal liberal use of topical skin formulations in the region. Fluoroquinolone and macrolide resistance are also common among PVL-positive clones, especially ST22 and ST80 variants. Alarmingly, resistance patterns in CA-MRSA now mirror those of traditional HA-MRSA.^7^ In Bahrain, there was no significant difference in resistance profiles between CA and HA strains.^8^ In Kuwait, PVL-positive CC22 clones exhibited resistance to five or more antibiotics in over half of isolates.^13^ Although vancomycin and linezolid remain effective, rising MICs and the spread of multi-drug-resistant clones warrant sustained vigilance.

### 4.4. Surveillance and laboratory gaps

Surveillance systems across the GCC are not fully developed. Most data come from research projects rather than structured national programs. This limits timely detection of emerging clones or shifts in resistance. Few clinical laboratories routinely test for PVL genes, and reliance on epidemiologic classification is increasingly unreliable due to the convergence of CA and HA strains. Advanced genotyping methods, including DNA microarrays and whole-genome sequencing, have provided key insights in select studies.^13^ However, these techniques remain confined to research centers. Scaling up molecular diagnostics and establishing reference labs for regional strain tracking would substantially strengthen MRSA control efforts.

### 4.5. One-Health implications

Although human-to-human transmission remains the primary route for PVL-MRSA, potential zoonotic or foodborne spillover cannot be excluded. Studies comparing patient and meat isolates identified limited PVL presence in food,^20,22^ but clonal overlaps, especially with ST5, ST6, and ST361, raise concerns about possible exchange between sectors. The discovery of PVL-positive MRSA in retail meat, combined with the identification of rare clones like CC7 and CC1153 with animal-related backgrounds, underscores the importance of integrated surveillance. Livestock contact, farming practices, and antimicrobial use in agriculture are potential drivers of resistance and clonal spread.

### 4.6. Public health and policy considerations

The convergence of virulence and resistance in MRSA strains circulating in the GCC poses significant challenges for infection control and clinical management. Hospitals must apply consistent precautions for all MRSA infections, regardless of presumed origin. Targeted screening, decolonization protocols, and environmental hygiene remain critical.

Antimicrobial stewardship is essential to prevent further resistance amplification. Empiric therapies must be guided by up-to-date susceptibility data, and overuse of topical antibiotics should be curtailed. The high rate of multidrug-resistant PVL-MRSA in community settings suggests a need for revising empirical treatment algorithms for skin and soft tissue infections.

### 4.7. Limitations

The included studies were substantially heterogeneous with respect to design, setting, sampling strategy, laboratory methods, and outcome reporting, limiting direct comparison across studies. No formal risk-of-bias appraisal was performed because this was a scoping review, and no meta-analysis was undertaken due to the marked heterogeneity of the available data. So, prevalence findings should be interpreted as study-level ranges rather than as a pooled regional estimate. The evidence base was also uneven across GCC countries, with underrepresentation of several settings and no eligible original studies identified from Oman, thereby limiting the geographic completeness of the review. Publication bias is also likely, as studies with more notable resistance or molecular findings may have been preferentially published. In addition, the inclusion of mixed study designs and a small number of preprint sources, where applicable, may have introduced further variability in the strength of the evidence.

## 5. CONCLUSION

This scoping review shows that PVL-positive MRSA has been frequently reported across several GCC settings, particularly in community-associated lineages, with occasional overlap into hospital-associated backgrounds. However, the evidence remains heterogeneous and incomplete, particularly for some countries. Strengthened regional surveillance, standardized molecular typing, and one-health-informed monitoring are needed.

## ACKNOWLEDGEMENT

The authors thank the Communicable Diseases Center, Hamad Medical Corporation, Doha, Qatar, for academic support and provision of institutional resources for data analysis. The authors also acknowledge the contribution of the HMC Medical Research Center’s Library Services and Qatar National Library for facilitating database access.

## AUTHOR CONTRIBUTIONS

SS: Conceptualization, study design, literature search, data extraction, meta-analysis, writing – original draft preparation, and overall supervision. ASO: Methodological review, critical revision for intellectual content, and validation of final analysis. TP: Data screening, extraction, and quality assessment. CP: Statistical analysis support, data visualization, and manuscript editing. MAM: Project oversight, interpretation of findings, and senior review of the manuscript. All authors reviewed and approved the final version of the manuscript and agreed to be accountable for all aspects of the work.

## CONFLICT OF INTEREST

The authors declare that they have no competing interests related to this work.

## DISCLOSURE OF AI USE

During the preparation of this manuscript, the authors used Claude, Anthropic; Opus 4.8 to assist with language editing and readability of author-written text. The tool was not used to generate scientific content, interpret data, or produce references. The authors reviewed and edited all output and take full responsibility for the content of the publication.

## DATA AVAILABILITY

All data generated or analyzed during this study are included in the published article and its supplementary materials. No new datasets were created.

## FUNDING

This study did not receive any specific grant from funding agencies in the public, commercial, or not-for-profit sectors.

## ETHICAL APPROVAL

As this article is a scoping review based exclusively on previously published data, it did not involve direct participation of human subjects or animals. Therefore, ethical approval and informed consent were not required. All data sources were properly cited, and the review was conducted in accordance with the PRISMA-ScR guidelines.

## Figures and Tables

**Table 1.** Characteristics of the included studies on Panton-Valentine leukocidin-positive methicillin-resistant *Staphylococcus aureus* in Gulf Cooperation Council countries.

ID	Study	Country/Setting	Sample
1	Boucherabine et al.^7^	UAE, multi-hospital	310 clinical MRSA isolates
2	Sharif et al.^9^	Saudi Arabia, Jeddah tertiary hospital	194 MRSA isolates from 175 patients
3	Alkuraythi et al.^20^	Saudi Arabia, Riyadh; clinical and retail meat	136 SA isolates: 85 clinical, 51 meat
4	Eltai et al.^17^	Qatar, 18 primary health centers	576 nasal and axillary swabs
5	Alkuraythi et al.^22^	Saudi Arabia, Riyadh; clinical and retail meat	84 clinical SA isolates and 250 meat samples yielding 53 SA
6	AlSaleh et al.^8^	Bahrain, tertiary hospital	161 SA isolates: 97 MRSA, 64 MSSA
7	Alsolami et al.^10^	Saudi Arabia, Ha’il tertiary hospitals	147 MRSA isolates
8	Alahmadi et al.^11^	Saudi Arabia, Medina hospital	83 MRSA isolates
9	Boswihi et al.^13^	Kuwait, 6 public hospitals	636 MRSA isolates, all CC22
10	Sarkhoo et al.^12^	Kuwait, 5 hospitals	182 MRSA isolates, all CC361
11	Monecke et al.^21^	UAE / Saudi Arabia within multi-country study	15 CC1153 MRSA and MSSA isolates
12	Tabaja et al.^18^	MENA regional data	Multiple studies, mixed sources
13	Mazi et al.^14^	Saudi Arabia, 3 hospitals	28 MRSA clinical isolates
14	Quadri et al.^16^	Saudi Arabia, hospital-based	71 MRSA clinical isolates

**Table 2.** Molecular epidemiology and PVL prevalence among included studies.

ID	Major Clone(s) / Lineage(s)	PVL Finding
1	CC5, CC6, CC22, CC1, CC8, CC30; USA300 detected	36%
2	Mainly CA-MRSA clones, including ST80 and ST30	60%
3	Human isolates diverse; meat isolates included ST398 and ST361	18% overall; 45% in clinical isolates
4	Not specified	0%
5	CC5, ST5, ST6; livestock-associated CC97 in meat	Not clearly reported
6	Mainly CA-MRSA; SCCmec IV and V common	62% in MRSA; 66% in CA-MRSA vs 33% in HA-MRSA
7	Mainly CA-MRSA lineages	Not directly reported
8	HA-MRSA background	0%
9	ST22-IV / CC22 predominated	About 21%
10	CC361 only	0%
11	CC1153	PVL present in all Gulf MRSA CC1153 isolates
12	CC5, CC8, CC22, CC30, CC80	PVL more frequent in CA-MRSA than HA-MRSA
13	ST22, mainly spa type t4573	46%
14	CA-MRSA with SCCmec IV / V; HA-MRSA with SCCmec I / II / III	50.7% overall; 67.5% in CA-MRSA vs 29% in HA-MRSA

**Table 3.** Antimicrobial resistance findings and main conclusions of included studies.

1	fusC, ermC, aacA-aphD detected; no vancomycin resistance
2	High resistance to clindamycin and erythromycin; vancomycin susceptible
3	Human MRSA is more resistant than meat isolates; erythromycin and clindamycin common
4	Resistance to ciprofloxacin, erythromycin, tetracycline, gentamicin, co-trimoxazole, and inducible clindamycin
5	SCCmec IV is more common in clinical MRSA; SCCmec V is more common in meat MRSA
6	Frequent fluoroquinolone and macrolide resistance; no vancomycin or linezolid resistance
7	Beta-lactam resistance universal; gentamicin, erythromycin, and fusidic acid resistance noted
8	Multidrug resistance common; vancomycin and linezolid remained active
9	Resistance to trimethoprim, ciprofloxacin, gentamicin, and erythromycin; hybrid SCCmec IV+V reported
10	fusC-mediated fusidic acid resistance common; aminoglycoside and ciprofloxacin resistance present
11	fusC and blaZ in all MRSA; tetK common
12	High resistance to oxacillin, erythromycin, and clindamycin; variable fusidic acid and co-trimoxazole susceptibility
13	PVL-positive ST22 isolates were ciprofloxacin resistant; vancomycin remained active
14	CA-MRSA is more likely to carry PVL than HA-MRSA; detailed resistance data limited

## References

[1] Al-Saleh A, Shahid M, Farid E, Bindayna K (2022). Trends in methicillin-resistant *Staphylococcus aureus* in the Gulf Cooperation Council countries: antibiotic resistance, virulence factors and emerging strains. East Mediterr Health J.

[2] Shoaib M, Aqib AI, Muzammil I, Majeed N, Bhutta ZA, Kulyar MF (2023). MRSA compendium of epidemiology, transmission, pathophysiology, treatment, and prevention within one health framework. Front Microbiol.

[3] Karmakar A, Jana D, Dutta K, Dua P, Ghosh C (2018). Prevalence of Panton-Valentine Leukocidin Gene among Community Acquired *Staphylococcus aureus*: A Real-Time PCR Study. J Pathog.

[4] Wannet WJ, Spalburg E, Heck ME, Pluister GN, Tiemersma E, Willems RJ (2005). Emergence of virulent methicillin-resistant *Staphylococcus aureus* strains carrying Panton-Valentine leucocidin genes in The Netherlands. J Clin Microbiol.

[5] Conly JM, Johnston BL (2003). The emergence of methicillin-resistant *Staphylococcus aureus* as a community-acquired pathogen in Canada. Can J Infect Dis.

[6] Loewen K, Schreiber Y, Kirlew M, Bocking N, Kelly L (2017). Community-associated methicillin-resistant *Staphylococcus aureus* infection: Literature review and clinical update. Can Fam Physician.

[7] Boucherabine S, Nassar R, Mohamed L, Habous M, Nabi A, Husain RA (2025). Methicillin-Resistant *Staphylococcus aureus*: The Shifting Landscape in the United Arab Emirates. Antibiotics (Basel).

[8] AlSaleh A, Shahid M, Farid E, Saeed N, Bindayna KM (2023). Multidrug-Resistant *Staphylococcus aureus* Isolates in a Tertiary Care Hospital, Kingdom of Bahrain. Cureus.

[9] Hala S, Fallatah O, Bahaitham W, Malaikah M, Alarawi M, Anasari H Concurrent Clonal Expansion of Community-Associated Methicillin-resistant *Staphylococcus aureus* (MRSA) Clones in a Tertiary Hospital 2024.

[10] Alsolami A, ALGhasab NS, Alharbi MSM, Bashir AI, Saleem M, Syed Khaja AS (2023). Community-Acquired Methicillin-Resistant *Staphylococcus aureus* in Hospitals: Age-Specificity and Potential Zoonotic-Zooanthroponotic Transmission Dynamics. Diagnostics (Basel).

[11] Alahmadi TFH, Alahmadey ZZ, Elbanna K, Neyaz LA, Ahmad I, Abulreesh HH (2023). The Prevalence and Clinical Characteristics of Multidrug-resistant Hospital-acquired *Staphylococcus aureus* in Medina, Saudi Arabia. J Pure Appl Microbiol.

[12] Sarkhoo E, Udo EE, Boswihi SS, Monecke S, Mueller E, Ehricht R (2021). The Dissemination and Molecular Characterization of Clonal Complex 361 (CC361) Methicillin-Resistant *Staphylococcus aureus* (MRSA) in Kuwait Hospitals. Front Microbiol.

[13] Boswihi SS, Verghese T, Udo EE (2022). Diversity of clonal complex 22 methicillin-resistant *Staphylococcus aureus* isolates in Kuwait hospitals. Front Microbiol.

[14] Mazi W, Alshammari F, Yu J, Alam MJ, Saeed M, Alshaghdali K, Saeed A (2020). A descriptive analysis of PVL-positive multidrug-resistant *Staphylococcus aureus* in hospital-associated infections in Saudi Arabia. Bioinformation.

[15] Alawad MJ, Zara S, Elgohari A, Ibrahim AS, Abdel Hadi H (2023). Catastrophic complications of PVL-MRSA necrotizing pneumonia presenting as respiratory failure and rhabdomyolysis, case report and review of the literature. Clin Case Rep.

[16] Quadri SA, Al-Sultan AA, Al-Ramdan AM, Badger-Emeka LI, Ali SI (2020). Frequency of Panton-Valentine Leukocidin Gene among Clinical Isolates of Methicillin-Resistant *Staphylococcus aureus* in Eastern Province of Saudi Arabia. J Glob Infect Dis.

[17] Nahla O, Eltai R., Kamar M., Salih K., Alawad M. (2024). Alex, Exploring MRSA Prevalence and Implications in Qatar’s Community. Journal of Global Antimicrobial Resistance.

[18] Tabaja H, Hindy JR, Kanj SS (2021). Epidemiology of Methicillin-Resistant *Staphylococcus Aureus* in Arab Countries of the Middle East and North African (MENA) Region. Mediterr J Hematol Infect Dis.

[19] Yamamoto T, Nishiyama A, Takano T, Yabe S, Higuchi W, Razvina O, Shi D (2010). Community-acquired methicillin-resistant *Staphylococcus aureus*: community transmission, pathogenesis, and drug resistance. J Infect Chemother.

[20] Alkuraythi DM, Alkhulaifi MM, Binjomah AZ, Alarwi M, Mujallad MI, Alharbi SA (2024). Comparative genomic analysis of antibiotic resistance and virulence genes in *Staphylococcus aureus* isolates from patients and retail meat. Front Cell Infect Microbiol.

[21] Monecke S, Müller E, Braun SD, Armengol-Porta M, Bes M, Boswihi S (2021). Characterisation of S. aureus/MRSA CC1153 and review of mobile genetic elements carrying the fusidic acid resistance gene fusC. Sci Rep.

[22] Alkuraythi DM, Alkhulaifi MM, Binjomah AZ, Alarawi M, Aldakhil HM, Mujallad MI (2023). Genetic characterization and dissemination of *Staphylococcus aureus* and *Staphylococci genus*: food and health perspective [Preprint]. bioRxiv.

